# Multidimensional assessment of urinary dysfunction and quality of life after cervical cancer treatment: a multicenter study integrating urodynamics and patient-reported outcomes

**DOI:** 10.3389/fonc.2026.1853725

**Published:** 2026-07-02

**Authors:** Krzysztof Bereza, Andrzej Kukiełka, Dominika Trojnarska, Beniamin Oskar Grabarek, Marcin Opławski, Tomasz Banaś

**Affiliations:** 1 Department of Mother and Child Health, Faculty of Health Sciences, Institute of Nursing and Midwifery, Jagiellonian University Medical College, Cracow, Poland; 2 Department of Radiation Oncology, University Hospital in Cracow, Cracow, Poland; 3 Department of Gynecology and Obstetrics with Gynecologic Oncology, Ludwik Rydygier Memorial Specialized Hospital, Cracow, Poland; 4 Department of Radiation Oncology, Ludwik Rydygier Memorial Specialized Hospital, Cracow, Poland; 5 Department of Radiotherapy, Affidea NU-MED Cancer Diagnostics and Therapy Centre, Zamosc, Poland; 6 Collegium Medicum, WSB University, Dabrowa Gornicza, Poland; 7 Department of Gynecology and Obstetrics, Faculty of Medicine and Health Sciences, Andrzej Frycz Modrzewski Kraków University, Krakow, Poland; 8 Department of Radiotherapy, Maria Sklodowska-Curie Institute-Oncology Centre, Cracow, Poland

**Keywords:** cervical cancer, quality of life, survivorship, urinary dysfunction, urodynamic

## Abstract

**Background:**

Cervical cancer treatment may result in long-term urinary dysfunction and impaired quality of life. However, studies integrating objective urodynamic findings with patient-reported outcome measures (PROMs) remain limited. This study evaluated urinary function, quality of life, sexual function, fatigue, and psychological well-being in women treated for cervical cancer, with particular emphasis on treatment modality.

**Methods:**

This multicenter observational study included 132 women, comprising patients treated with surgery alone (S; n = 15), surgery followed by adjuvant therapy (S+A; n = 53), definitive radiotherapy with or without chemotherapy (R; n = 26), and a control group without a history of gynecological malignancy (C; n = 38). Baseline assessments were performed before treatment initiation, with follow-up evaluations at 3, 6, and 9 months after treatment completion. Urogynecological evaluation included urodynamic testing and classification of urinary dysfunction, while patient-reported outcomes were assessed using the Incontinence Impact Questionnaire-7 (IIQ-7), Urogenital Distress Inventory-6 (UDI-6), Satisfaction With Life Scale (SWLS), Functional Assessment of Chronic Illness Therapy–Fatigue (FACIT-F), Hospital Anxiety and Depression Scale (HADS), and Female Sexual Function Index (FSFI).

**Results:**

Urinary dysfunction was a frequent complication and became more pronounced during follow-up, particularly among women receiving radiotherapy. The proportion of patients with normal bladder function progressively decreased, accompanied by an increase in overactive bladder and mixed urinary incontinence. Objective urodynamic findings generally indicated reduced bladder capacity, lower maximum urinary flow rate, increased post-void residual volume, and a higher prevalence of detrusor overactivity among women treated with radiotherapy. Pain intensity, fatigue, anxiety and depressive symptoms increased over time, whereas life satisfaction and sexual function deteriorated, particularly in patients receiving radiotherapy or combined treatment. Weak but statistically significant correlations were observed between urodynamic findings and patient-reported outcomes, suggesting limited concordance between objective and subjective measures of urinary dysfunction.

**Conclusions:**

Cervical cancer treatment, particularly radiotherapy, is associated with persistent urinary dysfunction and deterioration in quality of life, fatigue, psychological well-being, and sexual function. Combining urodynamic assessment with patient-reported outcomes provides a more comprehensive evaluation of treatment-related morbidity and may facilitate earlier identification of patients requiring supportive care.

## 1 Introduction

Cervical cancer remains one of the most common gynecological malignancies worldwide, despite significant advances in screening, vaccination, and treatment strategies (1). Improvements in early detection and multimodal therapy have led to increased survival rates, shifting clinical attention toward long-term outcomes and quality of life in survivors. As a result, understanding the functional consequences of treatment has become an essential component of comprehensive oncological care (2, 3).

Management of cervical cancer is highly dependent on disease stage. In patients with early-stage disease, treatment is primarily based on surgery, most commonly radical hysterectomy with pelvic lymph node assessment, with adjuvant radiotherapy or chemoradiotherapy administered when pathological risk factors are present. In contrast, for patients with locally advanced cervical cancer, definitive concurrent chemoradiotherapy followed by brachytherapy represents the standard of care and is associated with improved local control and survival outcomes (4, 5). Although these treatment strategies are effective in achieving disease control, both surgical and radiation-based approaches may adversely affect pelvic floor integrity, autonomic innervation, and lower urinary tract function, contributing to long-term urogenital morbidity (6, 7).

Urinary disorders following cervical cancer treatment encompass a broad spectrum of clinical presentations, including stress urinary incontinence, urgency urinary incontinence, overactive bladder symptoms, voiding dysfunction, and mixed forms of incontinence (8, 9). These disturbances are often multifactorial, resulting from surgical nerve injury, radiation-induced fibrosis, or the combined effects of multimodal therapy. Importantly, such symptoms may persist long after treatment completion and significantly impair daily Functioning (10, 11).

Beyond physical symptoms, urinary dysfunction has a profound psychosocial impact. It may limit social participation, reduce physical activity, and negatively influence emotional well-being, sexual health, and self-esteem. Consequently, the burden of disease extends far beyond traditional oncological outcomes, highlighting the importance of a holistic, patient-centered evaluation (12–14).

In recent years, validated patient-reported outcome measures (PROMs) have become increasingly important in both clinical practice and research, as they allow for a more patient-centered evaluation of treatment effects. Instruments such as the Incontinence Impact Questionnaire-7 (IIQ-7), the Urogenital Distress Inventory-6 (UDI-6), and the Female Lower Urinary Tract Symptoms (FLUTS) questionnaire enable a detailed and structured assessment of urinary symptoms and their impact on quality of life (15). When complemented by broader measures addressing general health status, fatigue, sexual function, and psychological well-being, these tools provide a multidimensional and clinically meaningful understanding of survivorship outcomes (16, 17).

However, despite the growing recognition of these issues, there remains a lack of integrated studies that simultaneously evaluate urodynamic findings, patient-reported urinary symptoms, and multidimensional quality-of-life outcomes in women treated for cervical cancer, particularly in relation to different therapeutic strategies (18).

Furthermore, the role of adjuvant therapy in exacerbating urinary dysfunction remains incompletely understood. While radiotherapy and chemoradiotherapy are known to contribute to tissue damage and functional impairment, their specific impact on urinary symptoms and patient-reported outcomes, compared with surgery alone, has not been sufficiently clarified in well-defined clinical cohort (8, 11, 19).

Therefore, the present multicenter study was designed to comprehensively evaluate urinary dysfunction and its association with quality of life, sexual function, fatigue, and psychological well-being in women treated for cervical cancer. The primary outcome was the prevalence and severity of urinary dysfunction, assessed using standardized urodynamic examinations and validated urinary symptom questionnaires (IIQ-7 and UDI-6). Secondary outcomes included quality of life (SWLS), fatigue (FACIT-F), anxiety and depression (HADS), sexual function (FSFI), and pain intensity (VAS).

We hypothesized that patients treated with radiotherapy, either alone or as part of multimodal therapy, would demonstrate a higher prevalence of urinary dysfunction and worse patient-reported outcomes compared with patients treated with surgery alone and women without a history of gynecological malignancy. Furthermore, we hypothesized that objective urodynamic abnormalities would correlate with patient-reported urinary symptoms and overall survivorship outcomes.

By comparing patients treated with surgery alone, surgery combined with adjuvant therapy, and radiotherapy-based treatment with a control group, we sought to provide a comprehensive and clinically relevant assessment of treatment-related functional impairment and its impact on long-term survivorship.

## 2 Material and methods

### 2.1 Study design and population

This multicenter observational study was conducted at three tertiary gynecologic oncology centers in Poland: the Department of Gynecology and Obstetrics with Gynecologic Oncology at Ludwik Rydygier Memorial Specialized Hospital in Kraków, the Department of Radiation Oncology at the University Hospital in Kraków, and the Department of Radiation Oncology at Ludwik Rydygier Memorial Specialized Hospital in Kraków. The study was designed to evaluate urinary dysfunction, quality of life, sexual function, fatigue, and psychological well-being in women treated for cervical cancer, with particular emphasis on the impact of different treatment modalities.

Participants were allocated to four groups according to clinical status and treatment modality. The control group (C) comprised women without a history of gynecological malignancy. Patients with cervical cancer were stratified into three treatment groups: surgery only (S), surgery followed by adjuvant therapy (S+A), and definitive radiotherapy with or without concurrent chemotherapy (R).

Patients were enrolled before initiation of oncological treatment and were prospectively followed for 9 months. Assessments of pain intensity (VAS), life satisfaction (SWLS), fatigue (FACIT-F), anxiety and depression (HADS), and sexual function (FSFI) were performed before treatment initiation and repeated at 3, 6, and 9 months after treatment completion. Urodynamic examinations and urinary symptom assessments using the IIQ-7 and UDI-6 questionnaires were conducted at 3, 6, and 9 months after treatment completion. At each follow-up visit, participants underwent gynecological examination and assessment of the study outcomes.

#### 2.1.1 Inclusion and exclusion criteria

Eligible participants were women aged ≥18 years with histologically confirmed cervical cancer who had completed surgical treatment, adjuvant therapy, or definitive radiotherapy according to current clinical guidelines. Patients were required to have no evidence of recurrent disease during follow-up and to provide complete clinical, urodynamic, and questionnaire data.

Exclusion criteria included pre-existing urinary incontinence or lower urinary tract dysfunction before cancer treatment, documented neurogenic bladder dysfunction, recurrent or metastatic disease during follow-up, previous urogynecological or pelvic floor surgery unrelated to cervical cancer treatment, previous pelvic exenteration or urinary diversion procedures, neurological disorders potentially affecting lower urinary tract function (including multiple sclerosis, Parkinson’s disease, spinal cord injury, stroke with persistent neurological deficits, and symptomatic lumbar disc disease with neurological impairment), uncontrolled diabetes mellitus associated with diabetic neuropathy, active urinary tract infection, severe psychiatric illness limiting questionnaire completion, and incomplete clinical, urodynamic, or questionnaire data. Patients who had undergone pelvic exenteration were excluded because of the substantial anatomical and functional alterations of the lower urinary tract associated with this procedure, which could significantly affect urinary outcomes and compromise the homogeneity of the study population. Women included in the control group had no history of gynecological malignancy, no diagnosed lower urinary tract disorders, no previous urogynecological surgery, and no known neurological conditions affecting bladder function.

### 2.2 Study groups

The control group consisted of women recruited from gynecological outpatient clinics who had no history of gynecological malignancy, pelvic radiotherapy, neurological disorders affecting lower urinary tract function, active urinary tract infection, or previously diagnosed urinary incontinence. Controls were selected to ensure comparability with the oncological cohort with regard to age, body mass index, and menopausal status. Detailed demographic and clinical characteristics of all study groups are presented in **Table 1** and summarized in the Results section.

**Table 1:** Baseline demographic and clinical characteristics of study and control groups.

Parameter	S (n=15)	S+A (n=53)	R (n=26)	C (n=38)
Age (years)	50.3 ± 10.2	51.8 ± 9.7	44.3 ± 12.2	52.1 ± 9.4
BMI (kg/m²)	25.9 ± 4.6	26.8 ± 4.9	25.4 ± 5.1	26.4 ± 4.3
Premenopausal, n (%)	8 (53.3%)	25 (47.2%)	16 (61.5%)	17 (44.7%)
Postmenopausal, n (%)	7 (46.7%)	28 (52.8%)	10 (38.5%)	21 (55.3%)
FIGO stage distribution	IA: 5 (33.3%)IB1: 8 (53.3%)IIA1: 2 (13.4%)	IA: 6 (11.3%)IB: 30 (56.6%)IIA: 17 (32.1%)	IB2: 4 (15.4%)IB3: 5 (19.2%)IIA2: 3 (11.5%)III: 10 (38.5%)IV: 4 (15.4%)	Not applicable
Histology – SCC, n (%)	11 (73.3%)	38 (71.7%)	19 (73.1%)	
Histology – Adenocarcinoma, n (%)	3 (20.0%)	12 (22.6%)	6 (23.1%)	
Histology – Adenosquamous, n (%)	1 (6.7%)	3 (5.7%)	1 (3.8%)	

Data are presented as mean ± standard deviation (SD) or number (percentage), as appropriate. S, surgery-only group; S+A, surgery plus adjuvant therapy group; R, radiotherapy group; C, control group; RT, radiotherapy; CT, chemotherapy; BMI, body mass index; FIGO, International Federation of Gynecology and Obstetrics; SCC, squamous cell carcinoma.

### 2.3 Urogynecological evaluation

Urodynamic evaluation was performed at 3, 6, and 9 months after treatment completion to objectively assess lower urinary tract function. The examination included uroflowmetry, post-void residual (PVR) assessment, filling and voiding cystometry, urethral profilometry, and measurement of Valsalva Leak Point Pressure (VLPP). All examinations were conducted at the Urodynamic Clinic of the Ludwik Rydygier Memorial Specialized Hospital in Kraków according to the recommendations of the International Continence Society (ICS) and the International Urogynecological Association (IUGA) (20, 21).

#### 2.3.1 Uroflowmetry and PVR assessment

The evaluation began with uroflowmetry using a standard uroflowmeter to assess urine flow rate, voided volume, and voiding time. A minimum voided volume of 150 mL was required for reliable interpretation. Maximum urinary flow rate (Qmax) was interpreted according to age- and sex-adjusted reference values. Following uroflowmetry, post-void residual urine volume was measured by ultrasound.

#### 2.3.2 Filling and voiding cystometry

Subsequently, multichannel urodynamic testing was performed with the patient positioned on a urological–gynecological chair. After local urethral anesthesia with lubricating gel, a transurethral bladder catheter and a rectal catheter were inserted to measure intravesical and intra-abdominal pressures. The pressure transducer system was calibrated to atmospheric pressure before each examination.

During filling cystometry, the bladder was filled with sterile saline and parameters including bladder sensation, compliance, cystometric capacity, and detrusor activity were recorded. Voiding cystometry was subsequently performed to assess bladder emptying and differentiate between impaired detrusor contractility and bladder outlet obstruction. Detrusor pressure (Pdet) was calculated using the following formula:


Pdet=Pves−Pabd

where Pves represents vesical pressure and Pabd represents abdominal pressure.

Electromyographic activity of the external urethral sphincter was additionally assessed using surface electrodes placed in the perianal region.

#### 2.3.3 Urethral profilometry and VLPP assessment

Urethral profilometry was performed to evaluate maximum urethral closure pressure, functional urethral length, and resting urethral pressure. Resting urethral pressures below 20 cm H_2_O were considered indicative of urethral sphincter dysfunction. Valsalva Leak Point Pressure (VLPP) was determined during standardized Valsalva maneuvers. VLPP was defined as the lowest intravesical pressure at which urinary leakage occurred in the absence of a detrusor contraction. A VLPP value above 60 cm H_2_O was considered supportive of stress urinary incontinence.

#### 2.3.4 Classification of urinary dysfunction

Based on clinical evaluation, urodynamic findings, and patient-reported symptoms, participants were classified into one of four diagnostic categories: no urinary dysfunction (NC), stress urinary incontinence (SUI), overactive bladder (OAB), or mixed urinary incontinence (MUI). SUI was diagnosed in patients presenting with involuntary urine leakage during physical exertion, coughing, or sneezing, supported by positive stress testing and/or urodynamic findings suggestive of urethral sphincter insufficiency. OAB was defined according to International Continence Society (ICS) criteria (20) as urinary urgency, usually accompanied by increased daytime frequency and nocturia, with or without urgency urinary incontinence. The diagnosis of OAB was established primarily on the basis of clinical symptoms. Cystometric evidence of detrusor overactivity, when present, was recorded as a separate urodynamic finding and was not required for the diagnosis of OAB. MUI was diagnosed when clinical features of both SUI and OAB were present. Patients without clinically significant urinary symptoms and without pathological urodynamic findings were classified as having no urinary dysfunction.

#### 2.3.5 Interpretation of urodynamic findings

All urodynamic studies were independently interpreted by two experienced investigators (K.B. and M.O.), both trained in urogynecology and urodynamic diagnostics. Interpretation was performed according to standardized ICS and International Urogynecological Association (IUGA) recommendations (20, 21). In cases of disagreement, the final classification was established through consensus review.

#### 2.3.6 Follow-up protocol

Assessments were conducted at four predefined time points: before treatment initiation (pretreatment) and at 3, 6, and 9 months after completion of treatment. Pain intensity (VAS), life satisfaction (SWLS), fatigue (FACIT-F), anxiety and depression (HADS), and sexual function (FSFI) were assessed at all four time points. In contrast, urodynamic evaluation and urinary symptom assessment using the IIQ-7 and UDI-6 questionnaires were performed only during follow-up visits at 3, 6, and 9 months after treatment completion, as these measures were intended to evaluate treatment-related urinary dysfunction. Participants with incomplete questionnaire data, missing clinical or urodynamic assessments, or loss to follow-up were excluded from the final analysis.

### 2.4 Incontinence impact questionnaire-7

The Incontinence Impact Questionnaire-7 (IIQ-7) is a validated short-form instrument derived from the original IIQ, designed to assess the impact of urinary incontinence on quality of life in women. It is widely used in both clinical practice and research settings. The questionnaire consists of seven items evaluating the influence of urinary incontinence on physical activity, daily responsibilities, mobility, social interactions, emotional well-being, sexual life, and overall sense of control. Each item is rated on a four-point scale ranging from no impact to significant impact. Higher total scores indicate a greater negative effect of urinary incontinence on quality of life.

### 2.5 Urogenital distress inventory-6

The UDI-6 is a shortened version of the Urogenital Distress Inventory, used to evaluate the severity of urinary symptoms and associated discomfort. The questionnaire includes six items addressing urinary frequency, nocturia, stress urinary incontinence, urgency urinary incontinence, difficulties in bladder emptying, and pain or discomfort during micturition. Each symptom is graded on a four-point scale from no problem to severe problem. Higher scores reflect greater symptom burden and may indicate the need for further diagnostic evaluation and therapeutic intervention.

### 2.6 Visual analog scale

Pain intensity was additionally assessed using the Visual Analog Scale (VAS). The VAS consists of a 10 cm line on which patients indicate their perceived pain intensity, with endpoints representing no pain and the worst imaginable pain.

### 2.7 Satisfaction with life scale

The Satisfaction With Life Scale (SWLS) is a psychometric tool used to measure global cognitive judgments of life satisfaction. It consists of five statements rated on a seven-point Likert scale, with total scores ranging from 5 to 35. Higher scores indicate greater life satisfaction, whereas lower scores reflect dissatisfaction or reduced well-being.

### 2.8 Functional assessment of chronic illness therapy – fatigue

The FACIT-F is a validated questionnaire designed to assess fatigue in patients with chronic illnesses. It includes 13 items evaluating the physical and functional impact of fatigue over the preceding week. Responses are rated on a five-point Likert scale, resulting in a total score ranging from 0 to 52. Lower scores indicate more severe fatigue, whereas higher scores correspond to better functional status.

### 2.9 Hospital anxiety and depression scale

The Hospital Anxiety and Depression Scale (HADS) is a self-administered questionnaire used to assess symptoms of anxiety and depression in clinical populations. It consists of 14 items divided into two subscales: anxiety (7 items) and depression (7 items). Each item is scored from 0 to 3, resulting in subscale scores ranging from 0 to 21. Scores of 0–7 indicate no clinically significant symptoms, 8–10 suggest subclinical levels, and scores ≥11 are indicative of clinically relevant anxiety or depression. The HADS is widely used for screening and monitoring emotional distress, particularly in patients with chronic or oncological conditions.

### 2.10 Female sexual function index

The Female Sexual Function Index (FSFI) is a validated multidimensional instrument used to assess sexual function in women. It comprises 19 items grouped into six domains: desire, arousal, lubrication, orgasm, satisfaction, and pain. Each domain contributes to a total score ranging from 0 to 36, with lower scores indicating greater sexual dysfunction. A total score below 26.55 is commonly used as a threshold for clinically significant sexual dysfunction. FSFI assessment was performed only among women who reported being sexually active at the respective study time point.

### 2.11 Statistical analysis

Statistical analyses were performed using StatPlus software (StatPlus Pro v7.3, AnalystSoft, Brandon, FL, USA). Continuous variables were expressed as mean ± standard deviation (SD), whereas categorical variables were presented as absolute numbers and percentages. All tests were two-sided, and a p value < 0.05 was considered statistically significant. Prior to inferential testing, the distribution of continuous variables was assessed using the Shapiro–Wilk test, and homogeneity of variances was verified using Levene’s test. Baseline comparisons between independent groups were performed using one-way analysis of variance (ANOVA) for continuous variables and chi-square tests for categorical variables. When significant overall differences were identified, Tukey’s honestly significant difference (HSD) test was used for *post hoc* pairwise comparisons. Longitudinal changes in questionnaire-derived outcomes were evaluated using repeated-measures ANOVA, with time treated as the within-subject factor and treatment group as the between-subject factor. *Post hoc* comparisons were performed when appropriate. Because all analyses were performed on participants with complete follow-up data and the study included a limited number of repeated measurements, repeated-measures ANOVA was selected as the primary analytical approach. The relatively small size of the surgery-only subgroup additionally limited the stability of more complex modeling strategies. Nevertheless, linear mixed-effects models may provide greater flexibility for handling repeated observations and potential confounding variables and should be considered in future studies with larger cohorts. Potential confounding variables, including age, body mass index, and menopausal status, were evaluated at baseline. As no statistically significant differences between groups were observed for these variables, adjustment for covariates was not incorporated into the primary analyses. Relationships between questionnaire-derived scores and urodynamic findings were assessed using Spearman’s rank correlation coefficient. For the FSFI questionnaire, analyses were restricted to sexually active participants at each assessment point. Therefore, sample sizes differed across time points and treatment groups. An *a priori* sample size analysis was performed using G*Power software (version 3.1, Heinrich Heine University, Düsseldorf, Germany) (22). For a one-way ANOVA comparing three independent groups, assuming a medium effect size (f = 0.25), a significance level of 0.05, and a statistical power of 80%, the required total sample size was estimated at 159 participants. The final study population included 132 women, which was below the initially estimated sample size. Therefore, the study may have been underpowered to detect small-to-moderate differences between groups, particularly in subgroup and longitudinal analyses.

## 3 Results

### 3.1 Baseline demographic and clinical characteristics of the study population

**Figure 1:**
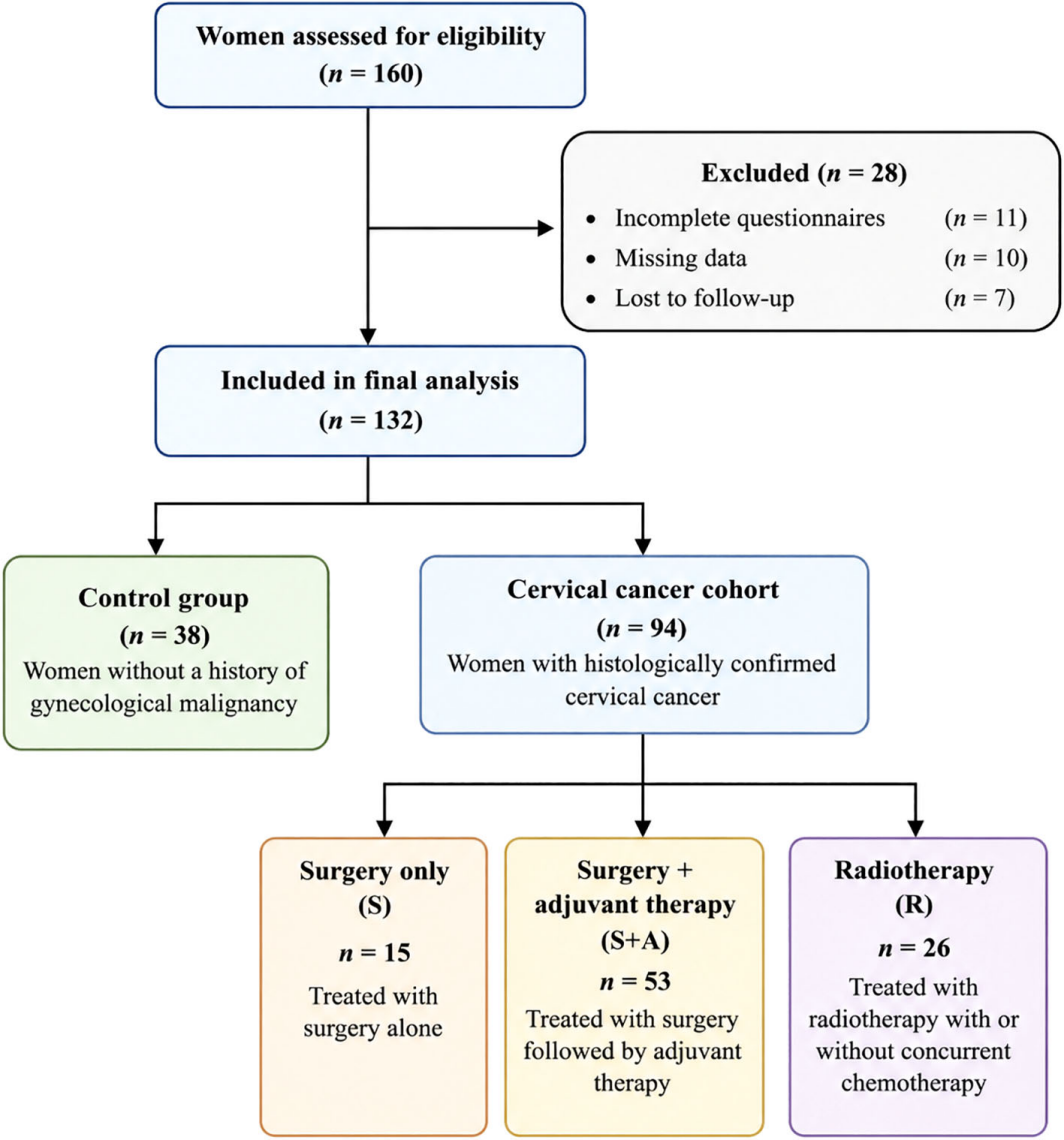
Flowchart of participant selection and allocation. A total of 160 women were screened for eligibility. Twenty-eight participants were excluded because of incomplete questionnaire data (n = 11), missing clinical or urodynamic data (n = 10), or loss to follow-up (n = 7). The final study population consisted of 132 women, including 94 patients treated for cervical cancer and 38 control participants. The cervical cancer cohort was stratified into surgery-only (S, n = 15), surgery plus adjuvant therapy (S+A, n = 53), and radiotherapy (R, n = 26) groups.

Participant recruitment and allocation are presented in **Figure 1**. A total of 160 women were screened for eligibility. Following exclusion of 28 participants due to incomplete questionnaire data (n = 11), missing clinical or urodynamic data (n = 10), or loss to follow-up (n = 7), 132 women were included in the final analysis, comprising 94 patients treated for cervical cancer and 38 control participants without a history of gynecological malignancy. Baseline demographic and clinical characteristics are summarized in **Table 1**. The study groups were comparable with respect to age, body mass index, menopausal status, and histological subtype distribution (all p > 0.05). Significant differences were observed only for FIGO stage distribution (p < 0.001), reflecting treatment allocation. Early-stage disease predominated in the S group, whereas patients in the R group generally presented with more advanced disease.

### 3.2 Urodynamic outcomes across treatment groups over time

Marked differences in urodynamic outcomes were observed between treatment groups at all evaluated time points (**Table 2**). Detailed objective urodynamic measurements, including Qmax, post-void residual volume, cystometric capacity, detrusor overactivity, VLPP, and functional urethral length, are presented in **Supplementary Table 1**. At 3 months, patients in the S group exhibited the highest proportion of NC, whereas the lowest proportion was observed in the R group (p = 0.041). This divergence became more evident over time. At 6 and 9 months, the proportion of patients without dysfunction decreased progressively, particularly in the R group (p = 0.028 and p = 0.019, respectively). Although individual categories of dysfunction (SUI, OAB, and MUI) did not differ significantly when analyzed separately, the overall pattern of urinary disturbances shifted toward OAB and MUI, especially among women treated with radiotherapy.

**Table 2:** Clinical classification of urinary dysfunction.

Time (months)	Result	S (n=15)	S+A (n=53)	R (n=26)	p-value*
3	NC	9 (60.0%)	22 (41.5%)	7 (26.9%)	0.041
	SUI	3 (20.0%)	12 (22.6%)	6 (23.1%)	
	OAB	2 (13.3%)	13 (24.5%)	7 (26.9%)	
	MUI	1 (6.7%)	6 (11.3%)	6 (23.1%)	
6	NC	8 (53.3%)	19 (35.8%)	5 (19.2%)	0.028
	SUI	3 (20.0%)	13 (24.5%)	6 (23.1%)	
	OAB	2 (13.3%)	14 (26.4%)	8 (30.8%)	
	MUI	2 (13.3%)	7 (13.2%)	7 (26.9%)	
9	NC	7 (46.7%)	17 (32.1%)	4 (15.4%)	0.019
	SUI	3 (20.0%)	13 (24.5%)	6 (23.1%)	
	OAB	3 (20.0%)	14 (26.4%)	9 (34.6%)	
	MUI	2 (13.3%)	9 (17.0%)	7 (26.9%)	

Data are presented as number (percentage). Comparisons between groups at each time point were performed using the chi-square test. P-values refer to differences in the overall distribution of urodynamic outcomes between groups within each time point. S, surgery-only group; S+A, surgery plus adjuvant therapy group; R, radiotherapy group; NC, no changes; SUI, stress urinary incontinence; OAB, overactive bladder; MUI, mixed urinary incontinence.

### 3.3 Impact of urinary symptoms on quality of life (IIQ-7)

Summary IIQ-7 scores across treatment groups and follow-up time points are presented in **Table 3**, whereas detailed item-level response distributions are provided in **Supplementary Table 1**. No statistically significant differences in mean IIQ-7 scores were observed between treatment groups at any analyzed time point (ANOVA, p > 0.05). Nevertheless, patients in the R group consistently demonstrated higher mean IIQ-7 scores than patients in the S and S+A groups throughout follow-up. The highest scores were observed in the R group at 3 and 9 months, indicating a greater perceived impact of urinary symptoms on daily functioning and quality of life. Detailed categorical analyses revealed a gradual shift toward higher symptom-burden categories over time, particularly among women treated with radiotherapy (**Supplementary Table 2**).

**Table 3:** Summary IIQ-7 and UDI-6 scores across treatment groups during follow-up.

Time (months)	IIQ-7 S	IIQ-7 S+A	IIQ-7 R	p-value	UDI-6 S	UDI-6 S+A	UDI-6 R	p-value
3	0.64 ± 0.83	1.48 ± 0.93	2.15 ± 0.86	>0.05	1.74 ± 1.10	1.16 ± 0.83	1.05 ± 1.00	>0.05
6	1.13 ± 0.73	1.28 ± 0.97	1.61 ± 0.93	>0.05	1.70 ± 1.06	0.53 ± 0.72	1.41 ± 0.99	>0.05
9	0.93 ± 0.71	1.08 ± 0.90	1.86 ± 1.04	>0.05	1.59 ± 1.15	0.80 ± 0.86	1.19 ± 1.03	>0.05

Values are presented as mean ± standard deviation (SD). Group comparisons at each time point were performed using one-way ANOVA. IIQ-7, Incontinence Impact Questionnaire-7; UDI-6, Urogenital Distress Inventory-6; S, surgery-only group; S+A, surgery plus adjuvant therapy group; R, radiotherapy group.

### 3.4 Severity of urinary symptoms (UDI-6)

Summary UDI-6 scores across treatment groups and follow-up time points are presented in **Table 3**, whereas detailed item-level response distributions are provided in **Supplementary Table 2**. No statistically significant differences in mean UDI-6 scores were identified between treatment groups at any follow-up assessment (ANOVA, p > 0.05). Although mean scores varied across groups and time points, patients in the R group generally exhibited higher symptom burden during follow-up than patients treated surgically. Detailed categorical analyses demonstrated a progressive increase in symptom severity, particularly in domains related to urgency and urinary leakage, with the most pronounced changes observed in the R group (**Supplementary Table 3**).

### 3.5 Correlation analysis and internal consistency of IIQ-7 and UDI-6

Correlation analysis was performed to assess the relationship between urodynamic findings and questionnaire-derived scores. For the IIQ-7 questionnaire, a weak but statistically significant positive correlation was observed with urodynamic outcomes (Spearman’s r = 0.12, p = 0.044). Similarly, for the UDI-6 questionnaire, a weak but statistically significant correlation was identified (r = 0.17, p = 0.0048), indicating a slightly stronger association compared to IIQ-7.

### 3.6 Pain intensity assessment using the VAS

Pain intensity assessed using the VAS is presented in **Table 4**, including *post-hoc* comparisons across groups and time points. At baseline, patients in all oncological groups demonstrated significantly higher VAS scores compared to the control group (p < 0.05). No significant differences were observed between treatment groups at this stage. At 3 months, a significant increase in pain intensity was observed in the R group compared to both S and S+A groups (p < 0.05). This trend persisted at 6 and 9 months, with consistently higher VAS scores in the R group. Within-group analysis revealed a significant increase in VAS scores over time in the R and S+A groups, whereas no statistically significant temporal changes were observed in the S group. At 9 months, the highest pain intensity was recorded in the R group, with significant differences observed both in intergroup comparisons and relative to earlier time points (p < 0.05).

**Table 4:** Pain intensity assessment in control and study groups.

Time point	Group	VAS score (Mean ± SD)
–	C	1.32 ± 0.81
Pretreatment	R	4.35 ± 1.52
	S	3.40 ± 1.76
	S+A	3.32 ± 1.37
3 months	R	6.00 ± 1.67
	S	3.27 ± 1.58
	S+A	4.06 ± 1.38
6 months	R	6.15 ± 1.05
	S	3.47 ± 1.64
	S+A	5.11 ± 1.27
9 months	R	7.04 ± 1.43
	S	3.20 ± 0.86
	S+A	5.15 ± 1.32

Values are presented as mean ± standard deviation. Overall differences between groups and time points were evaluated using repeated-measures ANOVA followed by Tukey’s *post-hoc* test.

Significant *post-hoc* comparisons (p < 0.05): all oncological groups vs control group; R vs S at all follow-up time points; R vs S+A at 6 and 9 months; significant increases from pretreatment values were observed in the R and S+A groups.

C, control group; S, surgery-only group; S+A, surgery plus adjuvant therapy group; R, radiotherapy group.

### 3.7 Quality of life assessment using the SWLS questionnaire

The results of the SWLS questionnaire are presented in **Table 5**. At 3 months after treatment, patients in the S group demonstrated higher SWLS scores than patients in the S+A and R groups. This pattern became more pronounced at 6 and 9 months, with the lowest scores consistently observed in the R group. Significant differences were identified between the control group and the oncological groups, as well as between the S and S+A groups (p < 0.05). Overall, life satisfaction declined over time, particularly among patients in the S+A and R groups.

**Table 5:** SWLS total score across treatment groups.

Group	Time point	Mean ± SD
C	Single assessment	27.61 ± 2.39
R	Pretreatment	22.42 ± 1.88
	3 months	17.58 ± 2.10
	6 months	15.35 ± 2.31
	9 months	15.23 ± 2.05
S	Pretreatment	24.73 ± 2.22
	3 months	22.33 ± 3.11
	6 months	20.13 ± 1.77
	9 months	20.00 ± 2.36
S+A	Pretreatment	24.13 ± 2.18
	3 months	20.38 ± 1.90
	6 months	18.40 ± 2.35
	9 months	17.79 ± 2.44

Values are presented as mean ± standard deviation. Overall differences between groups and time points were evaluated using repeated-measures ANOVA followed by Tukey’s *post-hoc* test.

Significant *post-hoc* comparisons (p < 0.05): all oncological groups vs control group; R vs S and S+A during follow-up; significant reductions from pretreatment values were observed in all treatment groups, with the greatest decline in the R group.

C, control group; S, surgery-only group; S+A, surgery plus adjuvant therapy group; R, radiotherapy group.

### 3.8 Fatigue assessment using the FACIT-F questionnaire

FACIT-F scores and *post-hoc* comparisons are presented in **Table 6**. At baseline, all oncological groups demonstrated significantly higher fatigue levels compared to the control group (p < 0.05). At 3 months, fatigue severity increased significantly in the R and S+A groups compared to the S group (p < 0.05), with the highest scores observed in the R group. At 6 and 9 months, fatigue levels continued to increase, particularly in the R and S+A groups, with statistically significant differences observed both between groups and within groups over time (p < 0.05). In contrast, the S group demonstrated a more moderate increase in fatigue, with relatively stable values between 6 and 9 months.

**Table 6:** FACIT-F fatigue score (0–52) across treatment groups.

Group	Time point	Mean ± SD
C	Single assessment	12.84 ± 4.15
R	Pretreatment	27.35 ± 5.12
	3 months	35.82 ± 4.98
	6 months	39.41 ± 5.36
	9 months	42.03 ± 5.01
S	Pretreatment	21.73 ± 4.88
	3 months	26.12 ± 5.34
	6 months	29.45 ± 4.21
	9 months	30.02 ± 4.66
S+A	Pretreatment	24.85 ± 5.01
	3 months	31.44 ± 4.77
	6 months	35.90 ± 5.22
	9 months	38.61 ± 5.43

Values are presented as mean ± standard deviation. Overall differences between groups and time points were evaluated using repeated-measures ANOVA followed by Tukey’s *post-hoc* test.

Significant *post-hoc* comparisons (p < 0.05): all oncological groups vs control group; R vs S at all follow-up assessments; R vs S+A at 6 and 9 months; significant increases in fatigue scores from pretreatment values were observed in all treatment groups.

C, control group; S, surgery-only group; S+A, surgery plus adjuvant therapy group; R, radiotherapy group.

### 3.9 Anxiety and depression assessment using the HADS questionnaire

The results of the HADS are presented in **Table 7**. At baseline, all oncological groups demonstrated significantly higher HADS scores compared to the control group (p < 0.05). No statistically significant differences were observed between the treatment groups at this stage. At 3 months, an increase in HADS scores was observed across all treatment groups, with significantly higher values in the R and S+A groups compared to the S group (p < 0.05). At 6 months, these differences became more pronounced, with further increases in HADS scores, particularly in the R group. Statistically significant differences were observed both between groups and within groups over time (p < 0.05). At 9 months, the highest HADS scores were recorded in the R group, followed by the S+A group, while the S group demonstrated relatively lower values. Intergroup differences remained statistically significant (p < 0.05). Within-group analysis revealed a progressive increase in HADS scores over time in the R and S+A groups, whereas the S group showed a more moderate increase, with no significant differences between 6 and 9 months.

**Table 7:** HADS total score across treatment groups with *post-hoc* comparisons.

Group	Time point	Mean ± SD
C	Single	9.12 ± 3.05
R	Pre	18.45 ± 4.12
	3 months	24.88 ± 3.95
	6 months	28.31 ± 4.22
	9 months	30.12 ± 4.05
S	Pre	14.22 ± 3.88
	3 months	17.55 ± 4.01
	6 months	19.43 ± 3.66
	9 months	19.88 ± 3.74
S+A	Pre	16.85 ± 3.91
	3 months	21.77 ± 3.82
	6 months	25.33 ± 4.11
	9 months	27.40 ± 4.36

Values are presented as mean ± standard deviation. Significant *post-hoc* comparisons (p < 0.05): all oncological groups vs control group; R vs S and S+A during follow-up; significant increases from pretreatment values were observed in all treatment groups.

### 3.10 Sexual function assessment (FSFI)

The results of the FSFI are presented in **Table 8**. FSFI analyses were performed exclusively among women who reported being sexually active at the respective assessment time point. Consequently, the number of participants included in the analysis varied across treatment groups and follow-up visits. A progressive reduction in the proportion of sexually active women was observed during follow-up, particularly in the S+A and R groups (**Table 8**). At baseline, all oncological groups demonstrated significantly lower FSFI scores compared to the control group (p < 0.05). No statistically significant differences were observed between treatment groups at this stage. At 3 months, a decrease in FSFI scores was observed across all treatment groups, with significantly lower values in the R and S+A groups compared to the S group (p < 0.05). At 6 months, this trend became more pronounced, with further reductions in FSFI scores, particularly in the R group. Statistically significant differences were observed both between groups and within groups over time (p < 0.05). At 9 months, the lowest FSFI scores were recorded in the R group, followed by the S+A group, while the S group maintained relatively higher values. Intergroup differences remained statistically significant (p < 0.05). Within-group analysis demonstrated a progressive decline in FSFI scores over time in the R and S+A groups, whereas the S group showed a more moderate decrease, with no significant differences between 6 and 9 months.

**Table 8:** FSFI total score (2–36) across treatment groups with *post-hoc* comparisons.

Group	Time point	Number of cases	Mean ± SD
C	single	38 (100%)	30.45 ± 3.12
R	pre	20 (77%)	22.35 ± 3.88
	3 months	16 (62%)	18.12 ± 3.45
	6 months	17 (65%)	15.44 ± 3.21
	9 months	17 (65%)	13.85 ± 3.10
S	pre	13 (87%)	26.18 ± 3.54
	3 months	8 (53%)	24.02 ± 3.91
	6 months	8 (53%)	22.45 ± 3.12
	9 months	8 (53%)	22.10 ± 3.33
S+A	pre	47 (89%)	24.85 ± 3.66
	3 months	27 (51%)	21.44 ± 3.28
	6 months	28 (53%)	18.95 ± 3.47
	9 months	28 (53%)	17.22 ± 3.59

Values are presented as mean ± standard deviation. Significant *post-hoc* comparisons (p < 0.05): all oncological groups vs control group; R vs S and S+A during follow-up; significant reductions from pretreatment values were observed in all treatment groups, with the lowest scores observed in the R group.

## 4 Discussion

The principal finding of this study is that women treated with radiotherapy, either alone or as part of multimodal treatment, experienced a greater burden of urinary dysfunction and less favorable patient-reported outcomes than women treated with surgery alone. These differences became increasingly apparent during follow-up and were accompanied by worsening pain, fatigue, psychological distress, and sexual dysfunction. Collectively, these observations suggest that treatment modality plays an important role in shaping functional recovery and quality of life following cervical cancer treatment (19, 23, 24). From a clinical perspective, the findings highlight the need for systematic long-term surveillance of urinary function in cervical cancer survivors, particularly among patients receiving radiotherapy. The progressive increase in urinary symptom burden observed during follow-up suggests that routine oncological care should be complemented by urogynecological assessment and early supportive interventions. Identification of patients at increased risk of urinary complications may facilitate timely implementation of pelvic floor rehabilitation, behavioral therapy, symptom-directed treatment, and psychosocial support, potentially improving long-term outcomes and quality of life.

Our results demonstrated that urinary dysfunction remained one of the most common treatment-related sequelae and that its prevalence varied according to therapeutic modality. These findings are consistent with previous reports indicating that radiation-based treatment is associated with greater impairment of lower urinary tract function than surgery alone (24–26). The progressive deterioration observed in the radiotherapy group is biologically plausible and may reflect chronic treatment-related fibrosis, microvascular injury, and alterations in neural pathways involved in bladder control, all of which may contribute to impaired storage and voiding function (10, 26–28).

An important observation of this study was the discrepancy between categorical and quantitative analyses of the IIQ-7 and UDI-6 questionnaires. Although aggregated questionnaire scores did not differ significantly between groups, categorical analyses demonstrated a gradual shift toward more severe symptom categories, particularly among patients receiving radiotherapy. This finding suggests that categorical response distributions may be more sensitive to subtle changes in symptom burden than composite summary scores. Alternatively, the relatively small sample size, especially in the surgery-only group, may have limited the ability to detect statistically significant differences in continuous outcomes. Therefore, the clinical significance of these findings should be interpreted cautiously.

Similarly, the correlations between urodynamic findings and questionnaire-derived outcomes were statistically significant but weak. The observed coefficients indicate limited concordance between objective measurements and patients’ subjective perceptions of urinary dysfunction. Rather than demonstrating a strong association, these findings suggest that urodynamic abnormalities and patient-reported symptoms represent related but distinct aspects of lower urinary tract dysfunction. Consequently, neither objective testing nor patient-reported outcomes alone appear sufficient to fully characterize the functional consequences of treatment.

Pain intensity increased over time, particularly among women treated with radiotherapy. Previous studies have suggested that chronic pelvic pain following oncological treatment may arise from several factors, including tissue fibrosis, inflammation, altered sensory processing, and treatment-related nerve injury (29–31).

Although causal relationships cannot be established within the present study, the parallel progression of pain and urinary dysfunction may indicate shared underlying mechanisms. Clinically, these observations support the need for integrated management approaches that address both urinary symptoms and chronic pain during survivorship care (32).

Fatigue, reduced quality of life, psychological distress, and sexual dysfunction followed a similar pattern, with the greatest deterioration observed among women receiving radiotherapy. Previous studies have demonstrated that these outcomes frequently coexist in cervical cancer survivors and may be influenced by treatment-related symptoms, chronic pain, concerns regarding disease recurrence, altered body image, and impaired physical functioning (33, 34). Rather than representing isolated complications, these factors may interact and contribute to long-term reductions in overall well-being. These observations support the need for multidisciplinary survivorship programs involving gynecologic oncologists, urogynecologists, physiotherapists, psychologists, pain specialists, and sexual health professionals.

The findings of the present multicenter study may have important implications for survivorship care in women treated for cervical cancer. Given the progressive increase in urinary dysfunction, pain intensity, fatigue, psychological distress, and sexual dysfunction observed during follow-up, particularly among patients receiving radiotherapy, a structured multidimensional follow-up strategy may be warranted. Such an approach could incorporate routine urogynecological assessment, including uroflowmetry and post-void residual volume measurements, together with validated patient-reported outcome measures evaluating urinary symptoms, quality of life, fatigue, and psychological well-being. Based on the temporal patterns observed in the present study, assessments performed at 3, 6, and 9 months after treatment completion may facilitate earlier identification of patients at risk of clinically significant treatment-related morbidity and allow timely implementation of supportive interventions. Nevertheless, this proposed follow-up framework should be regarded as hypothesis-generating and requires validation in prospective multicenter studies with larger patient populations before routine adoption in clinical practice.

The present study has several strengths. First, it employed a prospective multicenter design involving patients treated with different therapeutic modalities for cervical cancer. Second, both objective urodynamic assessments and validated patient-reported outcome measures were incorporated, enabling simultaneous evaluation of urinary function, quality of life, fatigue, psychological well-being, and sexual health. Third, repeated follow-up assessments at 3, 6, and 9 months allowed characterization of the temporal evolution of treatment-related morbidity. Finally, inclusion of a control group facilitated interpretation of the observed functional impairments within a broader clinical context.

Several limitations should also be acknowledged. First, baseline urodynamic assessments and urinary symptom questionnaires were not available before initiation of oncological treatment, limiting direct evaluation of treatment-induced changes in lower urinary tract function. Second, the treatment groups differed in size, with a relatively small number of patients in the surgery-only group, which may have reduced statistical power for selected subgroup analyses. Third, although the final cohort comprised 132 participants, the achieved sample size was lower than that estimated in the *a priori* power calculation, increasing the possibility of type II error for some outcomes. Fourth, the observational design introduces the possibility of residual confounding and selection bias despite comparable baseline demographic characteristics between groups. Although patients with pre-existing lower urinary tract dysfunction and major neurological disorders affecting bladder function were excluded, detailed information regarding all comorbidities potentially influencing urinary symptoms, including the severity of metabolic and neurological conditions, was not systematically analyzed. In addition, detailed obstetric and gynecological history, including parity, mode of delivery, fetal macrosomia, dystocic delivery, and previous pelvic floor rehabilitation, was not systematically collected. Consequently, the potential contribution of these factors to urinary outcomes could not be fully evaluated. Fifth, the follow-up period was limited to 9 months after treatment completion. Therefore, the present study may not capture late-onset urinary, sexual, or psychological complications that can emerge several years after cervical cancer treatment. The use of repeated-measures ANOVA instead of mixed-effects modeling may have limited the ability to account for within-subject variability and unbalanced group sizes. Because FSFI analyses were restricted to women who reported sexual activity at each assessment point, the results should be interpreted as reflecting sexual function among sexually active participants rather than sexual well-being in the entire cohort. This approach improves the validity of FSFI scoring but may introduce selection bias, as women who were sexually inactive because of treatment-related symptoms, pain, fatigue, psychological distress, lack of partner, or other factors were not included in the FSFI score analysis. Finally, although detailed clinical diagnostic categories and urodynamic findings were assessed, the study was not designed to investigate the biological mechanisms underlying urinary dysfunction. Accordingly, mechanistic interpretations regarding radiation-induced fibrosis, neural injury, or bladder remodeling should be considered hypothesis-generating and require confirmation in future studies.

Despite these limitations, the study provides clinically relevant evidence that urinary dysfunction and associated quality-of-life impairments vary according to treatment modality and may persist or worsen during survivorship, particularly among women receiving radiotherapy.

## 5 Conclusions

Urinary dysfunction represents a common complication following cervical cancer treatment and appears to be more pronounced among women receiving radiotherapy, either alone or as part of multimodal therapy. The observed urinary symptoms were accompanied by less favorable patient-reported outcomes, including increased pain, fatigue, psychological distress, and impaired sexual function. Although objective urodynamic findings demonstrated only weak correlations with patient-reported outcomes, the combined use of both assessment approaches provided a broader understanding of treatment-related morbidity. Furthermore, categorical analyses of urinary symptom questionnaires appeared to detect clinically relevant changes that were not consistently reflected in aggregated quantitative scores. These findings support the implementation of longitudinal monitoring and multidisciplinary survivorship care that incorporates urogynecological assessment, patient-reported outcome measures, psychological support, and rehabilitation strategies. Further prospective studies with larger cohorts, longer follow-up, and comprehensive baseline functional assessments are required to better characterize the long-term consequences of cervical cancer treatment and identify factors associated with adverse functional outcomes.

## Data Availability

The original contributions presented in the study are included in the article/**Supplementary Material**. Further inquiries can be directed to the corresponding author/s.
