# Correction: Multidimensional assessment of urinary dysfunction and quality of life after cervical cancer treatment: a multicenter study integrating urodynamics and patient-reported outcomes

**DOI:** 10.3389/fonc.2026.1929623

**Published:** 2026-07-20

**Authors:** Krzysztof Bereza, Andrzej Kukiełka, Dominika Trojnarska, Beniamin Oskar Grabarek, Marcin Opławski, Tomasz Banaś

**Affiliations:** 1 Department of Mother and Child Health, Faculty of Health Sciences, Institute of Nursing and Midwifery, Jagiellonian University Medical College, Cracow, Poland; 2 Department of Radiation Oncology, University Hospital in Cracow, Cracow, Poland; 3 Department of Gynecology and Obstetrics with Gynecologic Oncology, Ludwik Rydygier Memorial Specialized Hospital, Cracow, Poland; 4 Department of Radiation Oncology, Ludwik Rydygier Memorial Specialized Hospital, Cracow, Poland; 5 Department of Radiotherapy, Affidea NU-MED Cancer Diagnostics and Therapy Centre, Zamosc, Poland; 6 Collegium Medicum, WSB University, Dabrowa Gornicza, Poland; 7 Department of Gynecology and Obstetrics, Faculty of Medicine and Health Sciences, Andrzej Frycz Modrzewski Kraków University, Krakow, Poland; 8 Department of Radiotherapy, Maria Sklodowska-Curie Institute-Oncology Centre, Cracow, Poland

**Keywords:** cervical cancer, quality of life, survivorship, urinary dysfunction, urodynamic

The **Funding** statement was erroneously reported as no financial support had been received. The correct **Funding** statement is:

“The author(s) declare that financial support was received for the research and/or publication of this article. The article processing charge was funded by internal funds of the Jagiellonian University Medical College.”

The original version of this article has been updated.

