# Supplementary material for: Multidimensional assessment of urinary dysfunction and quality of life after cervical cancer treatment: a multicenter study integrating urodynamics and patient-reported outcomes
Source: Front Oncol. 2026 Jul 2;16:1853725. doi: 10.3389/fonc.2026.1853725 (PMC13372604; doi:10.3389/fonc.2026.1853725)
Supplement: Supplementary file 1 [file Table1.docx]

Supplementary Material

**Supplementary Table S1**. Detailed urodynamic parameters across treatment groups during follow-up.

| **Time (months)** | **Parameter** | **S** | **S+A** | **R** | **p-value** |
| --- | --- | --- | --- | --- | --- |
| 3 | Qmax (mL/s) | 24.8 ± 4.1 | 22.6 ± 4.5 | 19.7 ± 5.2 | 0.041 |
|  | PVR (mL) | 24 ± 12 | 39 ± 18 | 58 ± 24 | 0.032 |
|  | Cystometric capacity (mL) | 418 ± 62 | 396 ± 71 | 352 ± 75 | 0.028 |
|  | Detrusor overactivity (%) | 13.3 | 24.5 | 26.9 | 0.048 |
|  | VLPP (cm H₂O) | 82 ± 18 | 76 ± 21 | 72 ± 19 | 0.115 |
|  | Functional urethral length (cm) | 3.4 ± 0.4 | 3.2 ± 0.5 | 3.1 ± 0.5 | 0.221 |
| 6 | Qmax (mL/s) | 23.6 ± 4.5 | 21.4 ± 4.9 | 18.2 ± 5.4 | 0.027 |
|  | PVR (mL) | 28 ± 15 | 46 ± 22 | 69 ± 29 | 0.018 |
|  | Cystometric capacity (mL) | 404 ± 68 | 378 ± 73 | 331 ± 78 | 0.014 |
|  | Detrusor overactivity (%) | 13.3 | 26.4 | 30.8 | 0.021 |
|  | VLPP (cm H₂O) | 80 ± 19 | 74 ± 20 | 70 ± 21 | 0.126 |
|  | Functional urethral length (cm) | 3.3 ± 0.4 | 3.2 ± 0.5 | 3.0 ± 0.5 | 0.247 |
| 9 | Qmax (mL/s) | 22.8 ± 4.8 | 20.7 ± 5.1 | 16.8 ± 5.8 | 0.019 |
|  | PVR (mL) | 33 ± 17 | 54 ± 26 | 82 ± 35 | 0.011 |
|  | Cystometric capacity (mL) | 392 ± 71 | 362 ± 79 | 309 ± 82 | 0.009 |
|  | Detrusor overactivity (%) | 20.0 | 26.4 | 34.6 | 0.016 |
|  | VLPP (cm H₂O) | 79 ± 18 | 72 ± 22 | 68 ± 22 | 0.138 |
|  | Functional urethral length (cm) | 3.3 ± 0.5 | 3.1 ± 0.5 | 2.9 ± 0.6 | 0.261 |

Values are presented as mean ± standard deviation (SD) or percentage, as appropriate.

Abbreviations: S – surgery-only group; S+A – surgery plus adjuvant therapy group; R – radiotherapy group; Qmax – maximum urinary flow rate; PVR – post-void residual volume; VLPP – Valsalva Leak Point Pressure.

# Supplementary Table S2. Results of IIQ-7 questionnaire across treatment groups

| Time | Answer | Household | | | Physical | | | Entertainment | | | Travel | | | Social | | | Mental | | | Frustration | | |
| --- | --- | --- | --- | --- | --- | --- | --- | --- | --- | --- | --- | --- | --- | --- | --- | --- | --- | --- | --- | --- | --- | --- |
|  |  | S | S+A | R | S | S+A | R | S | S+A | R | S | S+A | R | S | S+A | R | S | S+A | R | S | S+A | R |
| 3 | No | 5 | 13 | 3 | 4 | 12 | 3 | 5 | 13 | 4 | 4 | 12 | 3 | 5 | 13 | 3 | 4 | 12 | 3 | 3 | 11 | 2 |
|  | Not at all | 4 | 11 | 4 | 4 | 11 | 4 | 4 | 10 | 4 | 3 | 10 | 4 | 4 | 11 | 4 | 4 | 10 | 4 | 3 | 9 | 3 |
|  | Some/A little | 3 | 14 | 7 | 3 | 14 | 7 | 3 | 13 | 7 | 3 | 14 | 7 | 3 | 13 | 7 | 3 | 14 | 7 | 3 | 14 | 7 |
|  | Medium | 2 | 9 | 6 | 2 | 9 | 6 | 2 | 9 | 6 | 2 | 9 | 6 | 2 | 9 | 6 | 2 | 9 | 6 | 2 | 10 | 6 |
|  | Very/A lot | 1 | 6 | 6 | 2 | 7 | 6 | 1 | 8 | 5 | 3 | 8 | 6 | 1 | 7 | 6 | 2 | 8 | 6 | 4 | 9 | 7 |
| 6 | No | 4 | 12 | 3 | 4 | 11 | 3 | 4 | 12 | 3 | 3 | 10 | 2 | 4 | 11 | 3 | 3 | 10 | 2 | 2 | 9 | 2 |
|  | Not at all | 4 | 10 | 4 | 4 | 10 | 4 | 4 | 10 | 4 | 3 | 10 | 4 | 4 | 10 | 4 | 4 | 10 | 4 | 3 | 9 | 4 |
|  | Some/A little | 3 | 13 | 7 | 3 | 13 | 7 | 3 | 13 | 7 | 3 | 13 | 7 | 3 | 13 | 7 | 3 | 13 | 7 | 3 | 13 | 7 |
|  | Medium | 2 | 10 | 6 | 2 | 10 | 6 | 2 | 9 | 6 | 2 | 10 | 6 | 2 | 10 | 6 | 2 | 10 | 6 | 2 | 11 | 6 |
|  | Very/A lot | 1 | 6 | 6 | 2 | 7 | 6 | 2 | 7 | 6 | 2 | 8 | 7 | 1 | 8 | 6 | 2 | 8 | 7 | 4 | 9 | 7 |
| 9 | No | 4 | 11 | 2 | 3 | 10 | 2 | 4 | 11 | 2 | 3 | 9 | 1 | 4 | 10 | 2 | 3 | 9 | 2 | 2 | 8 | 1 |
|  | Not at all | 4 | 10 | 3 | 4 | 9 | 3 | 4 | 10 | 3 | 3 | 9 | 3 | 4 | 9 | 3 | 4 | 9 | 3 | 3 | 8 | 3 |
|  | Some/A little | 3 | 12 | 7 | 3 | 13 | 7 | 3 | 12 | 7 | 3 | 13 | 7 | 3 | 12 | 7 | 3 | 13 | 7 | 3 | 13 | 7 |
|  | Medium | 3 | 12 | 7 | 3 | 12 | 7 | 2 | 11 | 7 | 3 | 12 | 7 | 3 | 12 | 7 | 3 | 12 | 7 | 3 | 12 | 7 |
|  | Very/A lot | 1 | 8 | 7 | 2 | 9 | 7 | 2 | 9 | 7 | 3 | 10 | 8 | 2 | 10 | 7 | 3 | 10 | 7 | 4 | 11 | 8 |

Values are presented as number of patients. S– surgery-only group; S+A – surgery plus adjuvant therapy group; R – radiotherapy group.

# Supplementary Table S3. Results of UDI-6 questionnaire across treatment groups

| Time | Answer | Frequency | | | Pressure leakage (R) | | | Stress leakage | | | Droplet leakage | | | Emptying problem | | | Pain (S) | | |
| --- | --- | --- | --- | --- | --- | --- | --- | --- | --- | --- | --- | --- | --- | --- | --- | --- | --- | --- | --- |
|  |  | S | S+A | R | S | S+A | R | S | S+A | R | S | S+A | R | S | S+A | R | S | S+A | R |
| 3 | No | 5 | 14 | 4 | 4 | 13 | 4 | 5 | 14 | 4 | 4 | 13 | 4 | 5 | 13 | 3 | 4 | 12 | 3 |
|  | Not at all | 4 | 11 | 5 | 4 | 11 | 5 | 4 | 10 | 5 | 4 | 11 | 5 | 4 | 11 | 5 | 4 | 10 | 5 |
|  | Some/A little | 3 | 14 | 7 | 3 | 14 | 7 | 3 | 13 | 7 | 3 | 14 | 7 | 3 | 13 | 7 | 3 | 14 | 7 |
|  | Medium | 2 | 9 | 6 | 2 | 9 | 6 | 2 | 9 | 6 | 2 | 9 | 6 | 2 | 9 | 6 | 2 | 9 | 6 |
|  | Very/A lot | 1 | 5 | 4 | 2 | 6 | 5 | 1 | 6 | 5 | 2 | 6 | 5 | 1 | 6 | 5 | 2 | 7 | 6 |
| 6 | No | 4 | 13 | 3 | 4 | 12 | 3 | 4 | 13 | 3 | 4 | 12 | 3 | 4 | 12 | 3 | 3 | 11 | 2 |
|  | Not at all | 4 | 10 | 4 | 4 | 10 | 4 | 4 | 10 | 4 | 4 | 10 | 4 | 4 | 10 | 4 | 4 | 10 | 4 |
|  | Some/A little | 3 | 13 | 7 | 3 | 13 | 7 | 3 | 13 | 7 | 3 | 13 | 7 | 3 | 13 | 7 | 3 | 13 | 7 |
|  | Medium | 2 | 10 | 6 | 2 | 10 | 6 | 2 | 9 | 6 | 2 | 10 | 6 | 2 | 10 | 6 | 2 | 10 | 6 |
|  | Very/A lot | 2 | 7 | 6 | 2 | 7 | 6 | 2 | 7 | 6 | 2 | 8 | 7 | 2 | 8 | 7 | 3 | 9 | 7 |
| 9 | No | 4 | 12 | 2 | 3 | 11 | 2 | 4 | 12 | 2 | 3 | 11 | 2 | 4 | 11 | 2 | 3 | 10 | 2 |
|  | Not at all | 4 | 10 | 3 | 4 | 9 | 3 | 4 | 10 | 3 | 4 | 9 | 3 | 4 | 9 | 3 | 4 | 9 | 3 |
|  | Some/A little | 3 | 12 | 7 | 3 | 13 | 7 | 3 | 12 | 7 | 3 | 13 | 7 | 3 | 12 | 7 | 3 | 13 | 7 |
|  | Medium | 3 | 11 | 7 | 3 | 11 | 7 | 2 | 11 | 7 | 3 | 12 | 7 | 3 | 11 | 7 | 3 | 12 | 7 |
|  | Very/A lot | 1 | 8 | 7 | 2 | 9 | 7 | 2 | 9 | 7 | 3 | 10 | 8 | 2 | 10 | 7 | 3 | 10 | 7 |

Values are presented as number of patients. Abbreviations: S – surgery-only group; S+A – surgery plus adjuvant therapy group; R – radiotherapy group.
